# Lightning-induced high temperature and pressure microstructures in surface and subsurface fulgurites

**DOI:** 10.1038/s41598-021-01559-x

**Published:** 2021-11-11

**Authors:** Li-Wei Kuo, Steven A. F. Smith, Chien-Chih Chen, Ching-Shun Ku, Ching-Yu Chiang, Dennis Brown, Marianne Negrini, Wen-Jeng Huang, Tze-Yuan Chen

**Affiliations:** 1 Department of Earth Sciences, National Central University, Taoyuan, 320 Taiwan; 2 Earthquake-Disaster & Risk Evaluation and Management Center, National Central University, Taoyuan, 320 Taiwan; 3 Department of Geology, University of Otago, 360 Leith Street, 9016 Dunedin, New Zealand; 4 National Synchrotron Radiation Research Center, Hsinchu, 30076 Taiwan; 5 Geosciences Barcelona, CSIC Barcelona, Barcelona, Spain; 6 Graduate Institute of Applied Geology, National Central University, Taoyuan, 320 Taiwan; 7 Present Address: Scientific Gear Service Co., Ltd, Hsinchu, 300039 Taiwan

**Keywords:** Geology, Mineralogy, Mineralogy

## Abstract

Cloud-to-ground lightning causes both high-temperature and high-pressure metamorphism of rocks, forming rock fulgurite. We demonstrate that a range of microstructural features indicative of high temperatures and pressures can form in fulgurites at the surface and in fractures up to several meters below the surface. In comparison to a granite reference sample collected from a borehole at a depth of 138 m, microstructures in both the surface and fracture fulgurite are characterized by: (i) the presence of glass, (ii) a phase transformation in K-feldspar with the presence of exsolution lamellae of plagioclase, and (iii) high residual stresses up to 1.5 GPa. Since this is the first time that fracture-related fulgurite has been described, we also carried out a 1-D numerical model to investigate the processes by which these can form. The model shows that the electric current density in fractures up to 40 m from the landing point can be as high as that on the surface, providing an explanation for the occurrence of fracture-related fulgurites. Our work broadens the near-surface environments in which rock fulgurite has been reported, and provides a detailed description of microstructures that can be compared to those formed during other types of extreme metamorphic events.

## Introduction

Lightning is a ubiquitous phenomenon on Earth (44 events per second on average^1^) which can dissipate up to 10^9^ J per flash^2^. In the vicinity of the landing point, cloud-to-ground lightning can cause shockwave pressures in excess of 10 GPa and temperatures above 1700 °C, resulting in high-temperature and high-pressure features being formed in the target material, which is sometimes converted into a sand or rock fulgurite (e.g.,^3–9^). Rock fulgurites are characterized by surface melting, and thin layers of a glassy surface crust are often described in the immediate vicinity of the landing point^8^. Based on this, it is typically assumed that the energy dissipated during a lightning strike is insufficient to develop lightning-induced features at distances of more than a few centimeters into a solid rock body^6^. However, the glassy rind of rock fulgurites is commonly weathered and altered, and thus they may be under-reported in comparison to sand fulgurites^6^. To date, there have been few comprehensive studies of the microstructural features formed in rock fulgurites during the transient high-temperature and high-pressure conditions experienced during a lightning strike^8,10–14^.

Here, we document in detail the composition and microstructural features of rock fulgurites that were formed by recent cloud-to-ground lightning events on Kinmen Island, Taiwan. This allows us to recognize and report the first known occurrence of rock fulgurite formed within fractures extending from the surface to a depth of several meters, suggesting that the extreme metamorphic effects of lightning are not restricted to the present-day topographic surface. Overall, our observations suggest that cloud-to-ground lightning can result in the formation of microstructures at high-temperature (> 1700 °C) and high-pressure (gigapascal-scale residual stress), both at the surface and along fractures to a depth of several meters in the granitic host rocks. Some of the microstructural and petrographic characteristics of the fulgurite samples described here are reminiscent of those reported to form during low-level shock metamorphism, and thus our observations also have consequences for the interpretation and distinction of extreme metamorphic events.

## Results

### Lightning occurrence and fulgurite formation on Kinmen Island, Taiwan

**Figure 1:**
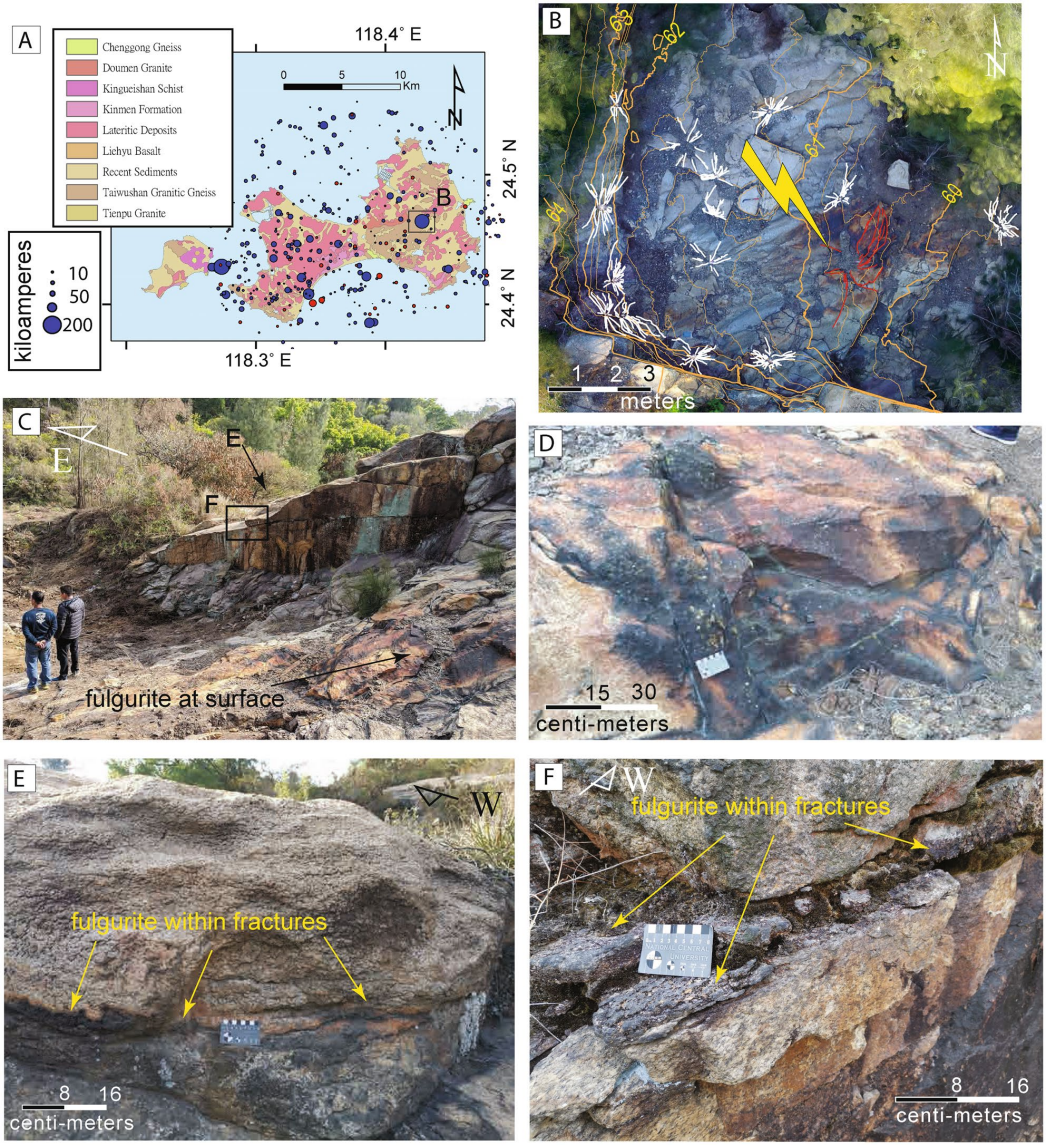
Geological setting of Kinmen Island, Taiwan, and the distribution of lightning on 7th May 2018. **(A)** Simplified geology of Kinmen Island, showing the distribution of the Taiwushan granitic gneiss. The map was created in QGIS version 3.4.14 (https://qgis.org). Individual CG lightning strikes and their energy are indicated by the location of size of circles. **(B)** Map view of the fulgurite collected by unmanned aerial vehicle with contours (24°27′51.6" N 118°25′37.9" E). **(C)** Outcrop photograph of two fulgurites including fresh brown-black glassy crust at the surface (surface fulgurite, part **(D)**) and within a shallowly-dipping fracture (fracture-related fulgurite; parts **(E,F)**). **(D)** Patch of fresh surface fulgurite around 1 m in diameter. **(E)** Fulgurite surrounding a shallowly-dipping fracture in granitic gneiss that is sub-parallel to the regional gneissic exfoliation joint. **(F)** Detail of fracture-related fulgurite observed on a subvertical outcrop face, showing a dark crust.

Kinmen Island, Taiwan, is comprised primarily of granitic rocks and their metamorphosed equivalents, mainly granitic gneiss^15^. These are unconformably overlain by Miocene and Pleistocene sedimentary rocks (Fig. 1a). The rock fulgurites described below are found on outcrops of weakly foliated granitic gneiss composed of c. 47% quartz, 43% feldspar (comprising K-feldspar and plagioclase), 7% biotite, and 3% accessory minerals (garnet, zircon, and magnetite;^15^). The gneissic fabric has a regional strike and dip of c. N4E 20E, and fractures are commonly developed sub-parallel to this fabric.

On 7th May 2018 more than 3000 lightning events were detected during a storm on Kinmen Island. About 14% of these were cloud-to-ground lightning (circles in Fig. 1a). The strike with the highest current intensity (~ 162 kiloamperes) came to ground on Mt. Taiwushan, the island’s highest mountain (253 m). Following the 7^th^ May storm, we traveled to Mt. Taiwushan in search of fulgurite. One fresh surface occurrence of fulgurite was identified, which occurs as a roughly 1-m diameter patch of brown-black glassy crust on the surface of granitic gneiss (Fig. 1b–d; “surface fulgurite” below). A wider examination of the outcrop also revealed a partially moss-covered fulgurite surrounding a shallowly-dipping fracture that intersects both the main sub-horizontal outcrop surface and several sub-vertical rock faces nearby (Fig. 1e,f; “fracture-related fulgurite” below). The fracture-related fulgurite lies sub-parallel to the regional gneissic foliation. At its lowest point, the fracture-related fulgurite is c. 2 m below the top surface of the outcrop. It is heterogeneously distributed along the fracture and variable in thickness between a few mm’s and a few cm’s. No indication of a recent lightning strike was found on the outcrop surface or on the vertical rock faces in the vicinity of the fracture-related fulgurite. Because of the covering of moss and the absence of a fresh glassy crust, we interpret the fracture-related fulgurite to have formed during an older cloud-to-ground lightning event. Since this is the first time that fracture-related fulgurite has been found, below we document its microstructures and then carry out 1D numerical modeling to investigate, a priori, whether or not it is possible to achieve the conditions needed to form the fulgurite we observe at depth along fractures.

### Microstructures of granitic gneiss reference sample

**Figure 2:**
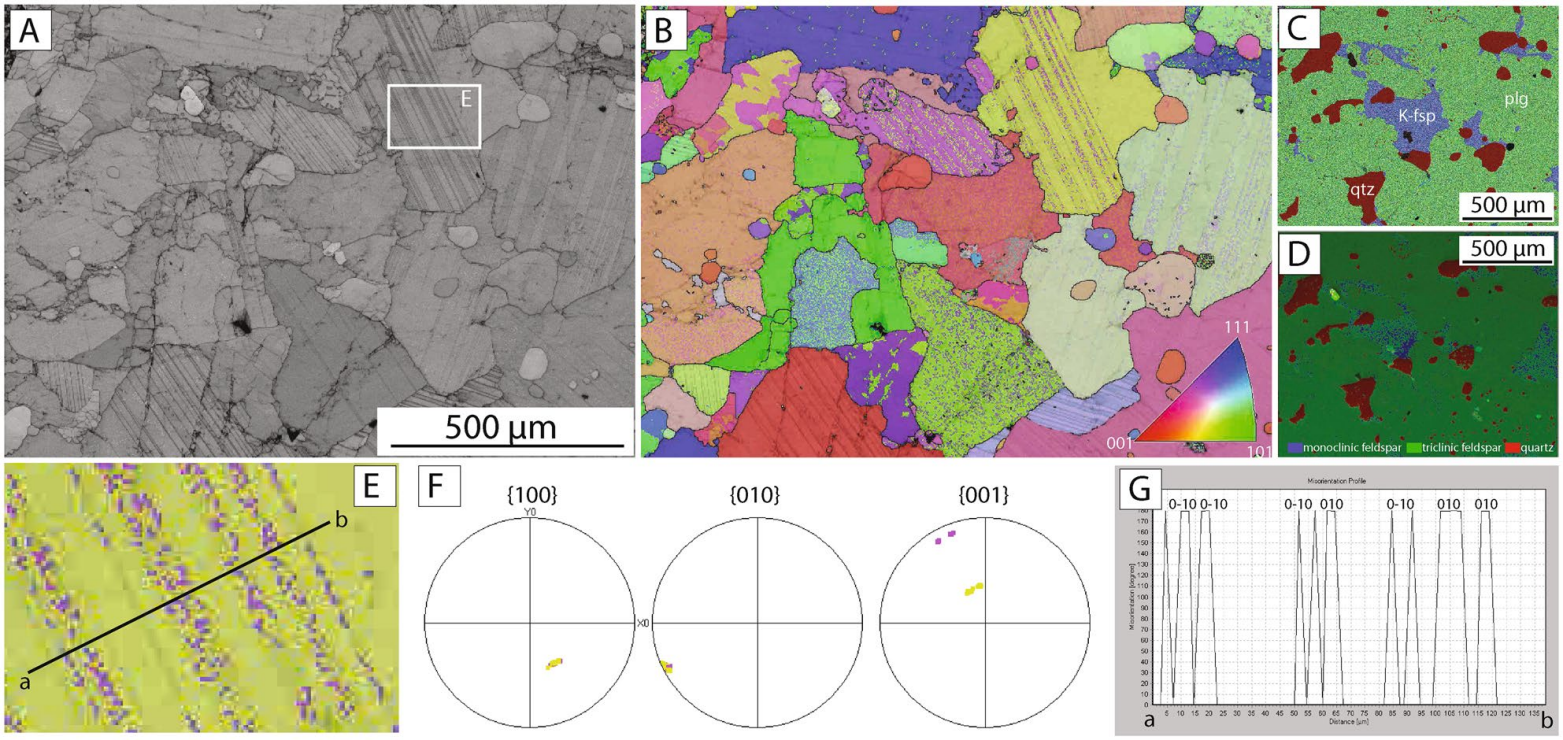
Crystallographic analysis of planar features in granitic gneiss reference sample. **(A)** Band contrast image acquired during EBSD analysis. Note the high density of twins in plagioclase grains and the absence of internal structures in K-feldspar. **(B)** Crystallographic orientation map colored according to the inverse pole figure color code (inset) for feldspar. Maps created by Oxford Instruments Aztec software and Oxford Instruments HKL Channel 5 software (https://nano.oxinst.com/products/aztec/aztechkl). **(C)** EDS chemical map and **(D)** EBSD phase map shows the distribution of mineral phases in the sample. Quartz is red, plagioclase is green, and K-feldspar is blue. **(E–G)** Crystallographic analysis of a plagioclase grains (location of analysis indicated in image **(A)**). **(E)** EBSD orientation map overlaid on band contrast image. Black line shows the location of the misorientation profile shown in **(G)**. **(F)** Pole figures (upper hemisphere) show the orientation of (100), (010) and (001) axes of the plagioclase crystal. **(G)** Misorientation profile across the examined grains shows the rotation angle (180°) and rotation axis.

The reference sample, collected from a depth of 138 m in the Mt. Taiwushan, contains plagioclase (c. 80%), K-feldspar (c. 10%) and quartz (c. 10%) (Fig. 2a–c). Representative chemical compositions of the feldspars are presented in Table 1. Plagioclase grains are 200–500 μm in size and have irregular boundaries with other phases (Fig. 2a,b). Well-defined sets of planar twins up to 50 μm wide cut across plagioclase grains from one side of the grain to the other (Fig. 2a,e). Electron backscatter diffraction (EBSD) analysis carried out along profiles across plagioclase twins indicates a consistent rotation angle of 180˚ around (010) (Fig. 2g). Twins are formed parallel to the (010) plane and perpendicular to (100) and (001) (Fig. 2f). K-feldspar grains are irregular in shape but do not show internal deformation or other types of internal structure (e.g., twins, exsolution lamellae) (Fig. 2a–c). EBSD analysis indicates that all K-feldspar grains have monoclinic crystal symmetry, while all plagioclase grains are triclinic (Fig. 2d). Quartz is present as irregular to subrounded grains up to 200 μm in size (Fig. 2a–d). The quartz commonly shows undulose extinction and evidence for internal distortion, including the development of subgrains, suggesting limited intracystalline plasticity (Fig. 2b). Late-stage brittle fractures cut across multiple grains (Fig. 2a) but do not alter the original microstructures and chemical composition of the granitic gneiss reference sample.

**Table 1:** Chemical compositions (oxide %) of feldspar grains and exsolution lamellae from the three samples described in the text.

	Na	Al	Si	K	Ca	Total
**Granite reference**						
K-Feldspar	0.99	19.23	63.26	15.69		99.17
Plagioclase	9.81	22.28	64.97	0.47	2.33	99.86
**Surface fulgurite**						
K-Feldspar	0.84	19.36	63.86	15.82		99.87
Exsolution lamellae	8.02	20.47	67.36	4.11		99.97
Exsolution lamellae	11.06	20.64	68.52	0.24		100.45
Plagioclase	10.18	22.22	65.29	0.22	2.07	99.98
**Fracture fulgurite**						
K-Feldspar	1.06	19.48	63.64	15.51		99.69
Exsolution lamellae	7.25	20.18	66.39	6.13		99.94
Plagioclase	10.02	22.33	65.33	0.36	2.11	100.15

### Microstructures of surface fulgurite

**Figure 3:**
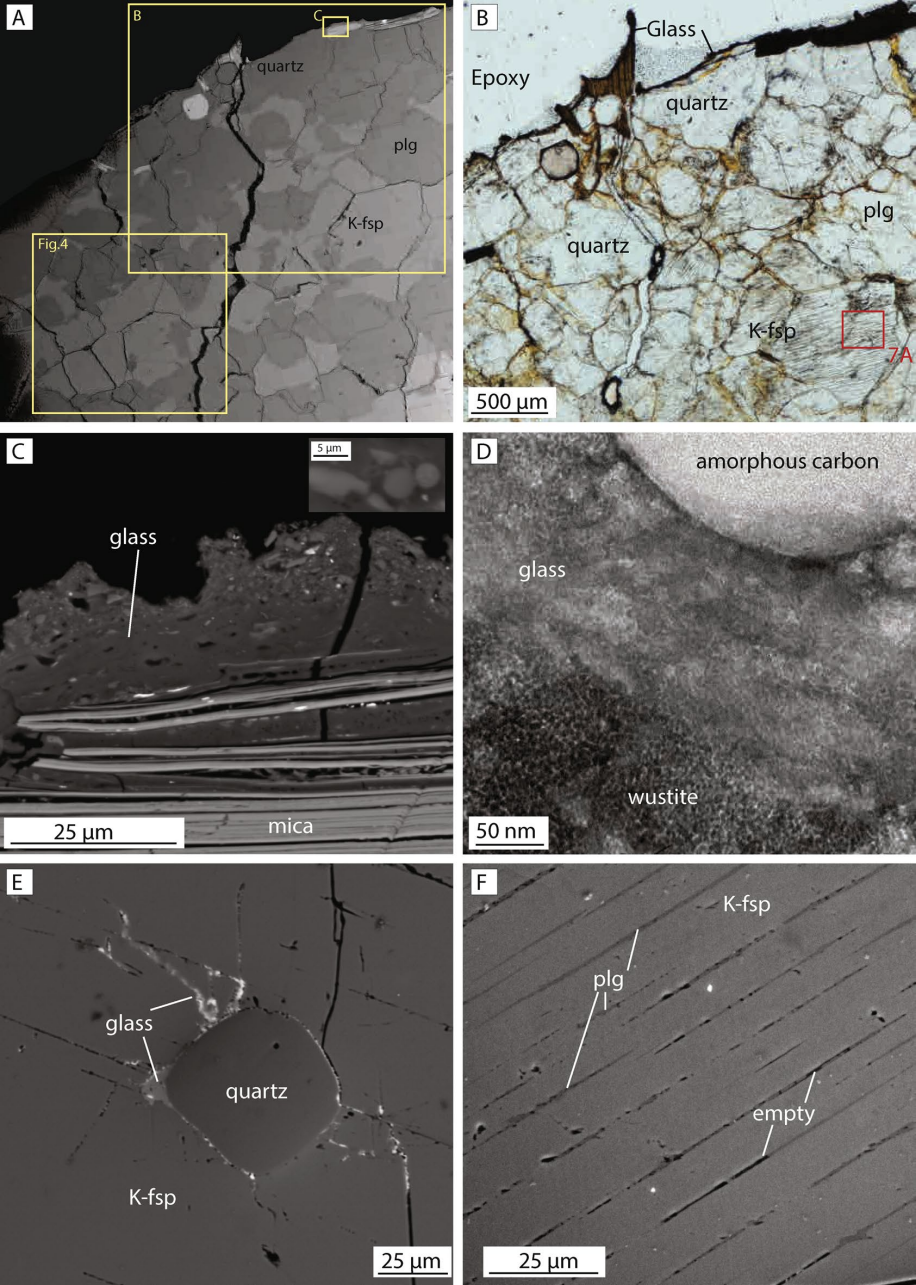
Microstructural characteristics of surface fulgurite sample. **(A)** Overview SEM backscatter image shows the distribution of quartz and plagioclase and K-feldspar, and the locations of images (**B,C** and Fig. 4). **(B)** Optical microscope image (plane polarized light) shows the surface glass and underlying quartz and K-feldspar with planar exsolution lamellae. Location of in-situ synchrotron Laue diffraction analysis is indicated by red box **(C)** SEM image shows the glassy surface crust and infiltration of glass along the cleavage surfaces of biotite. **(D)** Transmission electron microscope brightfield images show the coexistence of granular wüstite and amorphous carbon in an amorphous matrix in the fulgurite on the surface. Only the amorphous glassy matrix (in the limited observed area) was observed in the fracture-related fulgurite. **(E)** Thin layer of Si-rich glass forming around a quartz grain enclosed in K-feldspar with exsolution lamellae. **(F)** Detail of parallel, closely spaced planar structures found in K-feldspar, interpreted in the text as exsolution lamellae of plagioclase.

The surface fulgurite is characterized by a 10–100 μm thick glassy crust that overlies an up to one-centimeter-thick zone of fractured quartz and feldspar grains (Fig. 3a,b). In Scanning electron microscope (SEM) images, the glassy crust contains porous blebs in which round, reduced-oxidation-state iron oxides and inclusions are found (Fig. 3c,d). Under Focused ion beam–transmission electron microscopy (FIB-TEM), the iron oxide grains (wüstite) are found to be ~ 5 nm in size and the inclusions to be amorphous carbon (Fig. 3c,d), which may be related to lightning-induced reduction of the oxidation state^16^. Locally where the glass abuts biotite grains, it appears to infiltrate biotite along cleavage planes (Fig. 3c). At distances of up to 1 mm from the glassy surface, thin layers and pockets of incipient Si-rich glass are observed around the edges of rounded quartz grains in contact with plagioclase, and the glass locally injects along planar fractures in the feldspar (Fig. 3e).

**Figure 4:**
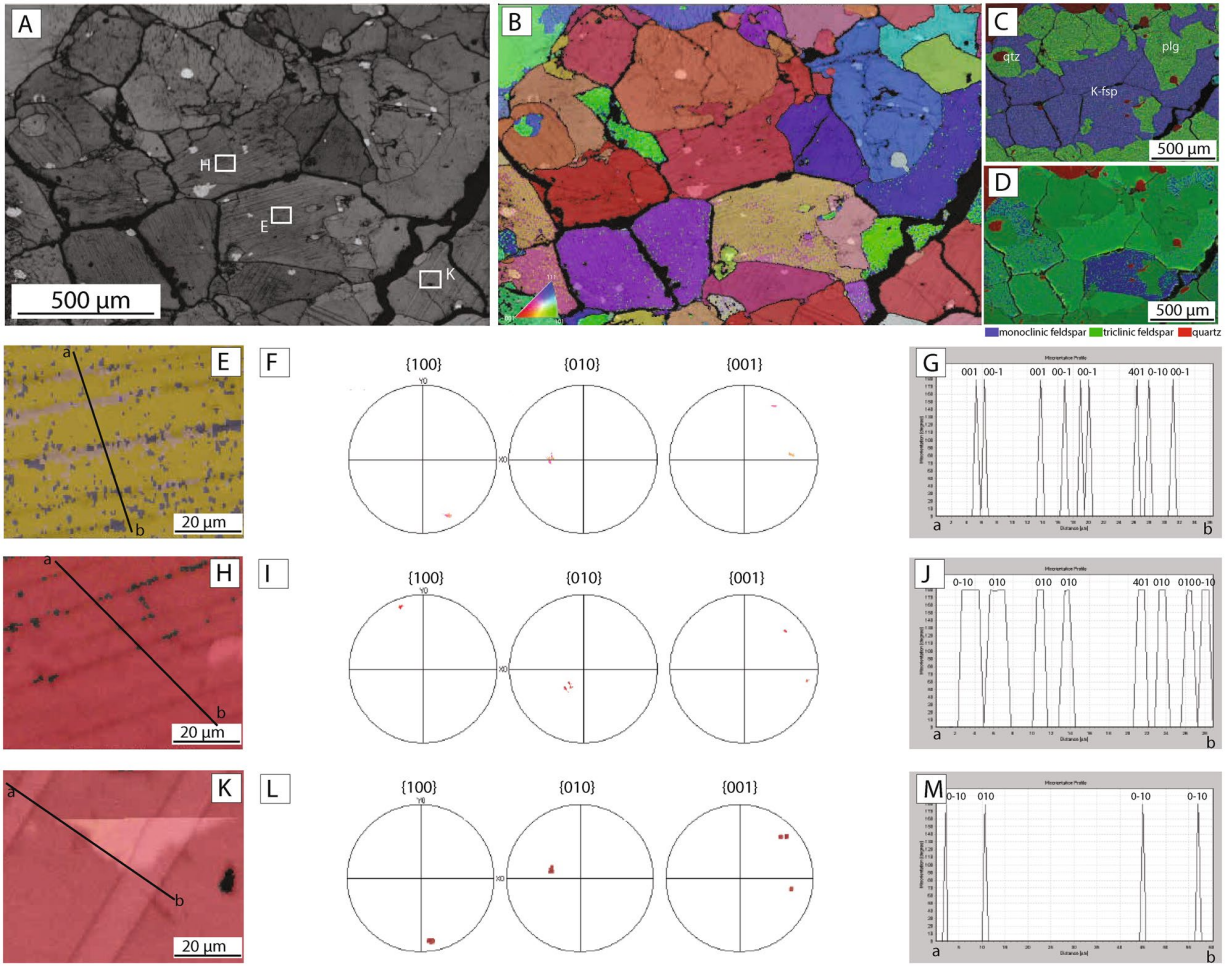
Crystallographic analysis of the surface fulgurite. **(A)** Band contrast image acquired during EBSD analysis. Note the presence of planar features in most of the K-feldspar grains (both monoclinic and triclinic K-feldspar **(D)**, but absence of planar features in the plagioclase (except for a few twins in the upper left corner). **(B)** Crystallographic orientation map colored according to the inverse pole figure color code for feldspar (inset). **(C)** EDS chemical map. Quartz is shown in red, plagioclase is green and K-feldspar is blue. **(D)** EBSD phase map shows the distribution of mineral phases in the sample. Quartz is shown in red, triclinic feldspar (Plagioclase) as well as triclinic K-feldspar is green and monoclinic feldspar (K-feldspar) is blue. Note that some K-feldspar grains have monoclinic crystal symmetry while others have a triclinic one. **(E–M)** Crystallographic analysis of a monoclinic K-feldspar **(E–G)**, a triclinic K-feldspar **(H–J)**, a triclinic Plagioclase **(K–M)**. The locations of the analysis are indicated in image **(A)**. **(E,H,K)** Orientation map overlaid on band contrast image. Black line shows the location of the misorientation profile shown in **(G,J,M)**. **(F,I,L)** Pole figure (upper hemisphere) shows the orientation of (100), (010) and (001) axes of the crystals. **(G,J,M)** Misorientation profile across the examined grains shows the rotation angle and rotation axis.

A widely developed microstructural characteristic of the surface fulgurites (and the fracture-related fulgurites described below) is the presence of sets of parallel planar structures in nearly all K-feldspar grains (Figs. 3f, 4). The planar structures are spaced at distances of between 5 and 25 μm and are between 20 and 300 μm long (Figs. 3f, 4a). However, they are not continuous across entire grains but instead terminate within grains such that the outer rim of K-feldspar grains is largely devoid of planar features (Fig. 4a). Although some of the planar features appear empty, most are filled by a 1–5 μm thick layer with different chemical compositions to the surrounding K-feldspar host grains (Fig. 3f). Electron dispersive spectroscopy (EDS) point analyses indicate that the material along the planar structures has a plagioclase composition (Table 1). On this basis, we interpret the sets of planar features to be plagioclase exsolution lamellae in K-feldspar host grains and refer to these features below as exsolution lamellae.

EBSD analysis (Fig. 4) indicates that some K-feldspar host grains containing exsolution lamellae maintain their original monoclinic crystal symmetry, while others have transformed to a triclinic crystal structure (Fig. 4d). Crystallographic analysis of both monoclinic and triclinic K-feldspars shows that the exsolution lamellae are developed subparallel to the (100)-plane in the host grain (Fig. 4e–j). Plagioclase feldspar grains in the surface fulgurite occasionally contain twins with similar characteristics to the granitic reference sample (Fig. 4k–m), although the abundance of twins is much lower than in the granite reference sample (Figs. 3a, 4a). Plagioclase grains do not contain exsolution lamellae or other types of planar features (Fig. 4a–c). 

### Microstructures of fracture-related fulgurite

**Figure 5:**
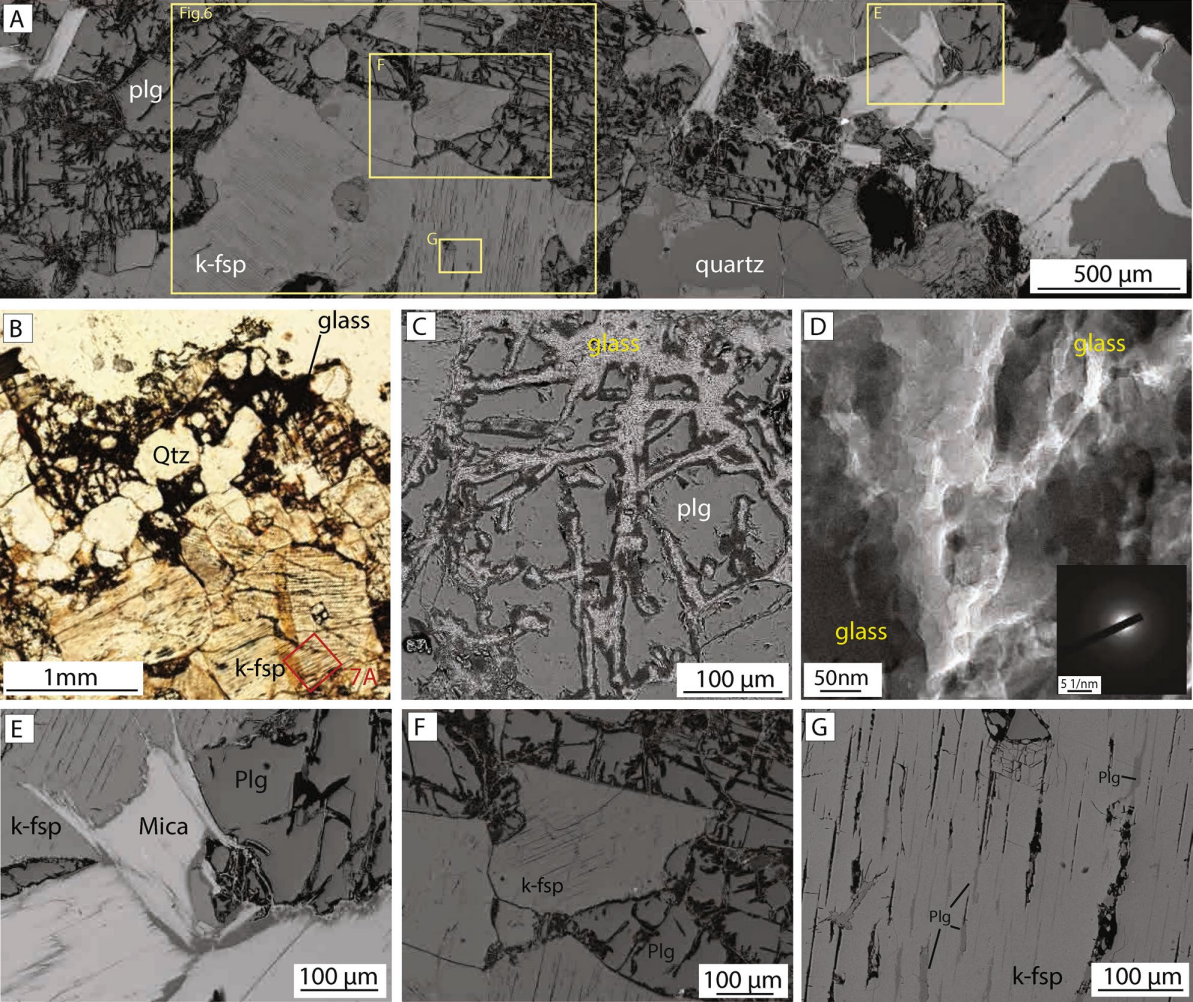
Microstructural characteristics of fracture-related fulgurite sample. **(A)** SEM backscatter image overview of the examined sample. Note the highly fractured (and altered) plagioclase where glass networks have formed, and the relative absence of glass in K-feldspar grains. **(B)** Optical microscope image (plane polarized light, shows the distribution of glass networks along grain boundaries. Location of in-situ synchrotron Laue diffraction analysis indicated by red box. **(C)** Backscatter image shows the glass network in plagioclase. **(D)** Transmission electron microscope brightfield image shows amorphous nature of the glassy fulgurite matrix. **(E)** Detailed backscatter image of the glass film surrounding the biotite and crosscutting plagioclase grains. **(F,G)** Backscatter images of exsolution lamellae formed in the k-feldspar host grains. Note the homogeneous nature and absence of internal structures in the plagioclase grains in **(F)**.

**Figure 6:**
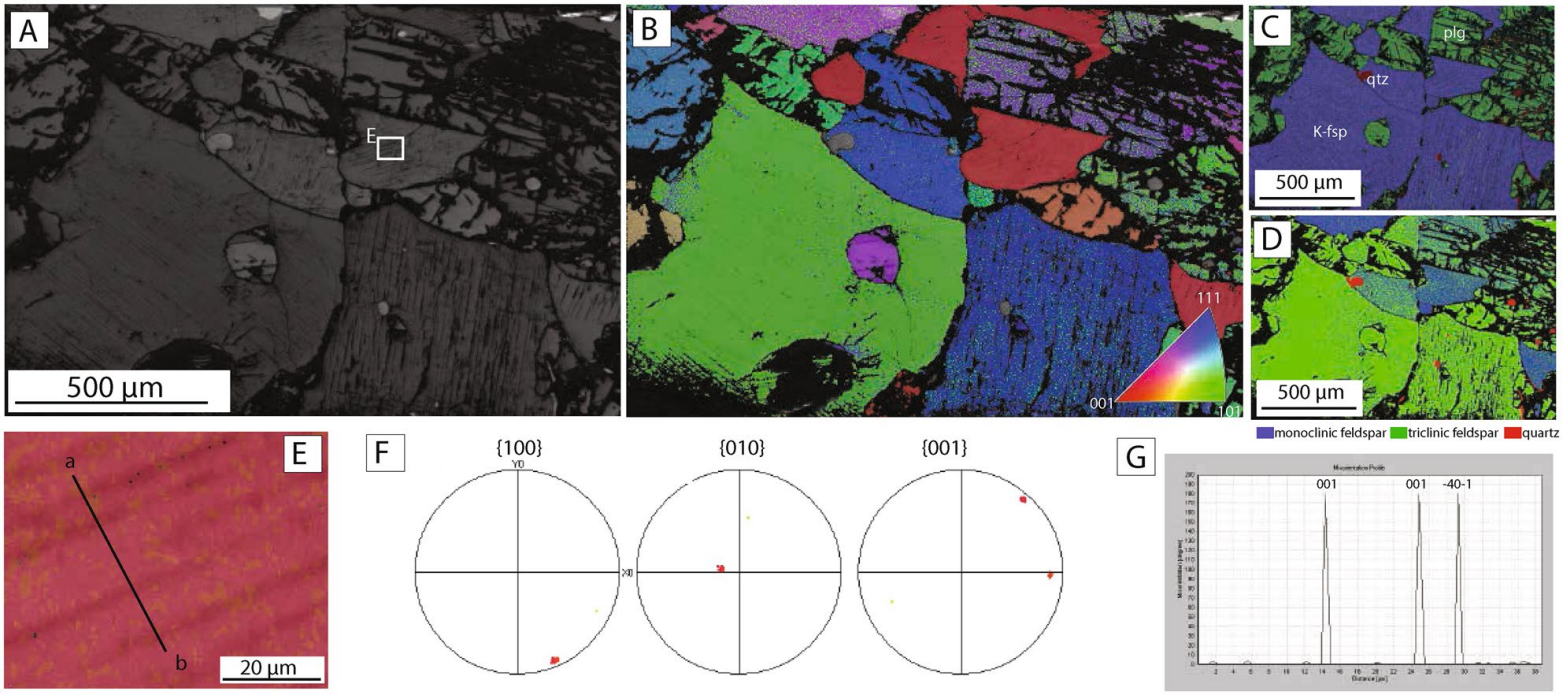
Crystallographic analysis of feldspar in the fracture-related fulgurite sample. **(A)** Band contrast image acquired during EBSD analysis. Note the presence of prominent planar features in the K-feldspar and the absence of internal structures in the plagioclase. **(B)** Crystallographic orientation map colored according to the inverse pole figure color code for feldspar (inset). **(C)** EDS chemical map. Quartz is shown in red, plagioclase is green, and K-feldspar is blue **(D)** EBSD phase map shows the distribution of mineral phases in the sample. Quartz is shown in red, triclinic feldspar (Plagioclase) as well as triclinic K-feldspar is green and monoclinic feldspar (K-feldspar) is blue. Note that some K-feldspar grains have monoclinic crystal symmetry while others have a triclinic one. **(E–G)** Crystallographic analysis of a monoclinic K-feldspar. **(E)** Orientation map overlapped on band contrast image. Black line shows the location of the misorientation profile shown in **(G)**. **(F)** Pole figure (upper hemisphere) shows the orientation of (100), (010) and (001) axis of the K-feldspar crystal. **(G)** Misorientation profile across the examined grains shows the rotation angle and rotation axis.

The fracture-related fulgurite contains angular to rounded fragments of heavily fractured quartz, K-feldspar and plagioclase within an optically opaque glassy matrix (Fig. 5a–c). The glass forms irregular and complex networks that penetrate along grain boundaries and fractures (Fig. 5a,b). The glass is found mainly in association with plagioclase and biotite grains (Fig. 5a,e), although it is also present along grain boundaries of quartz and K-feldspar (Fig. 5b,c). FIB-TEM and EBSD analyses indicate that the matrix is amorphous (Figs. 5d, 6a–d), similar to the glassy crust developed in the surface fulgurite.

Exsolution lamellae of plagioclase are widely developed within K-feldspar host grains and have similar characteristics to those developed within the surface fulgurite (Figs. 5f, g, 6). The exsolution lamellae are contained mainly within the central regions of large K-feldspar grains (Fig. 5f). The lamellae containing plagioclase are slightly more irregular than in the surface fulgurites, and often show serrated margins with the host grains (Fig. 5g). They are always formed subparallel to the (100)-plane in the host K-feldspar grain (Fig. 6f). As in the surface fulgurite, K-feldspar grains have monoclinic and triclinic symmetry (Fig. 6d) and plagioclase grains do not show any twins (Fig. 6a,b).

### Residual stress in K-feldspar, plagioclase and quartz from Laue diffraction analysis

**Figure 7:**
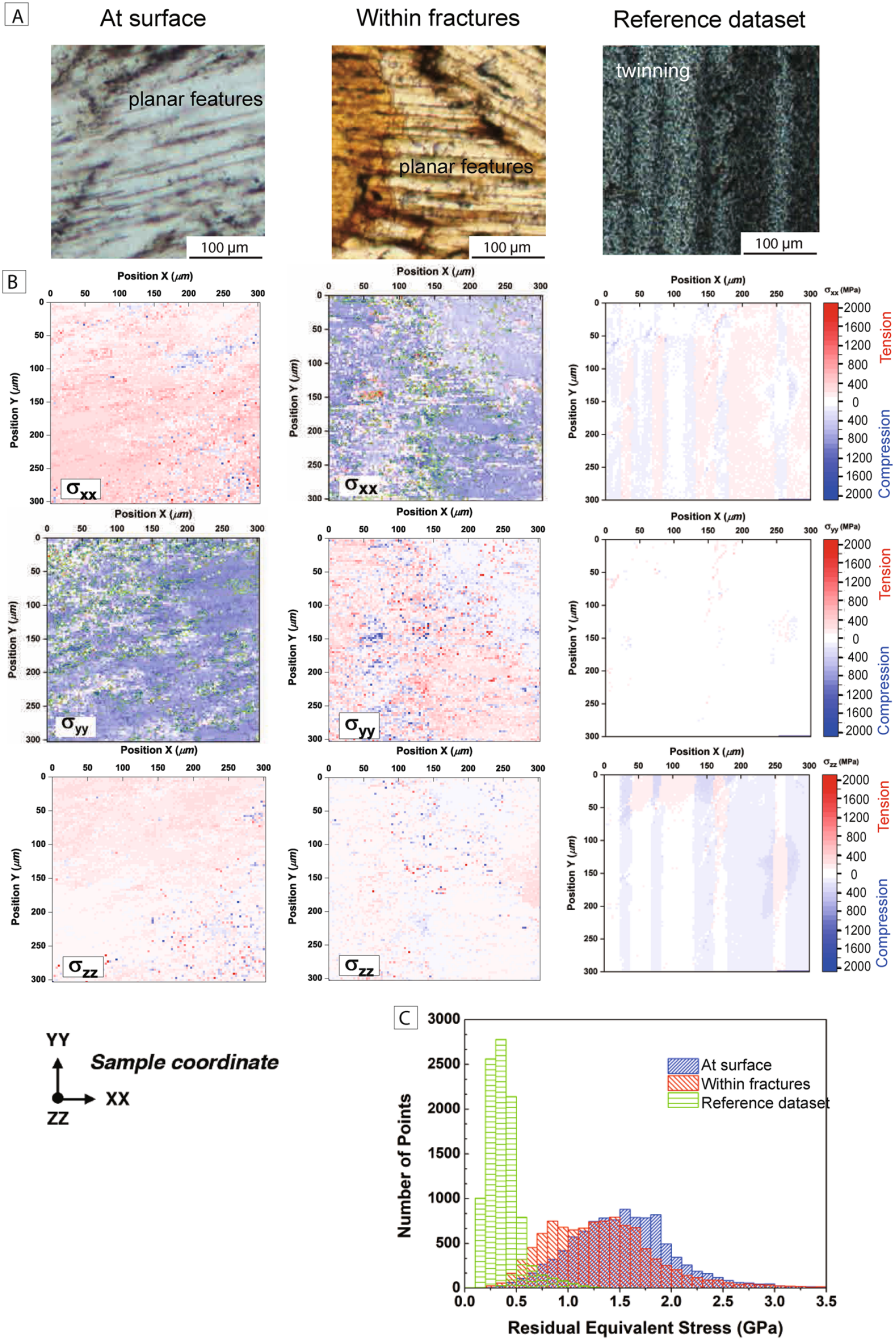
Residual stress mapping and the statistics of residual stress in K-feldspar and plagioclase from the two fulgurites and the reference sample (from a depth of 138 m). **(A)** Optical microscope images (plane polarized light images for two fulgurites and cross polarized image for granitic gneiss reference dataset) show planar features (exsolution lamellae) in K-felspar and twinning in plagioclase, respectively. **(B)** Distribution and magnitudes of axial stresses σ_xx_, σ_yy_, and σ_zz_ in K-feldspar and plagioclase. The gigapascal-scale residual deviatoric stress was determined with some exceptions that were higher than 2 GPa (green-circled area) within the K-feldspar from the two fulgurites. Plagioclase containing residual stress values of 0.38 ± 0.13 MPa on average in different stress direction and parallel to the intrinsic twining. **(C)** Statistics of residual stresses from the two fulgurites and the reference sample.

Calculations derived from Laue diffraction analyses (Fig. 7) indicate that the average residual stress in K-feldspar grains (sanidine) from the surface fulgurite is ~ 1.57 ± 0.20 GPa, ~ 1.39 ± 0.13 GPa within K-feldspar from fracture-related fulgurites. The calculated average residual stress in quartz grains, adjacent to the analyzed K-feldspar grains, is ~ 0.50 ± 0.07 GPa and ~ 0.38 ± 0.05 GPa for surface and fracture-related fulgurites, respectively (Supplementary Fig. S1). Because of the abundance of plagioclase grains in the reference sample, the data of Laue diffraction on plagioclase could be set as a representative regional stress at depth. At-depth plagioclase contains ~ 0.38 ± 0.06 GPa from the granitic reference sample (Fig. 7c). In the two fulgurites, the direction of the major residual stress (either σ_xx_ or σ_yy_ direction) is sub-parallel to the exsolution lamellae, whereas the direction of lowest residual stress is perpendicular to the exsolution lamellae, along the σ_zz_ direction (Fig. 7b). Synchrotron Laue diffraction mosaic mapping with a spatial resolution of 50 nm carried out on K-feldspar grains from the two fulgurites shows that the exsolution lamellae are subparallel to the (100) crystal plane (Fig. 7), consistent with results from EBSD analysis.

### Current density distribution from numerical modeling

**Figure 8:**
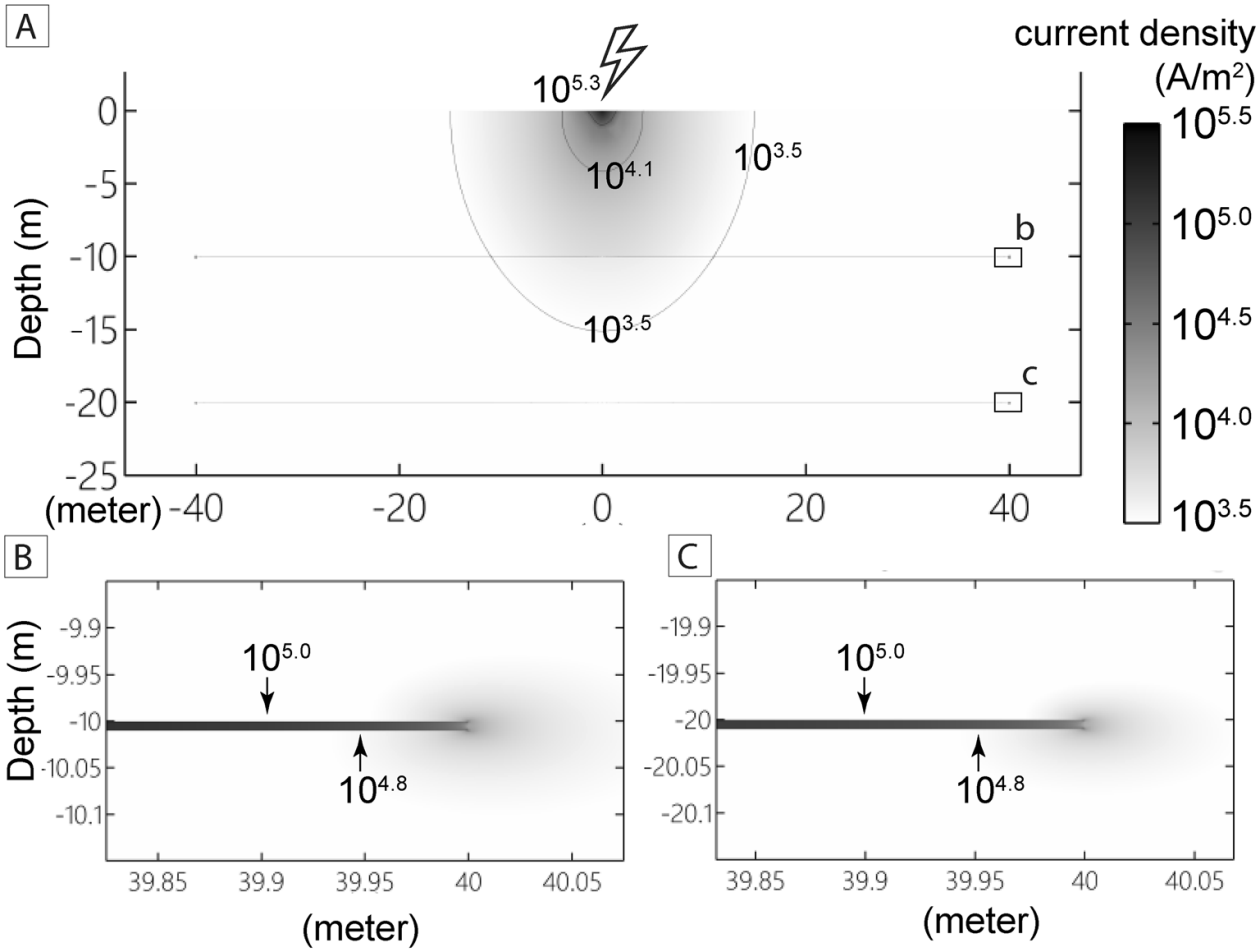
Numerical modeling of energy distribution during a lightning strike. **(A)** At the landing point, lightning injects a strong electrical current that diffuses into the homogeneous granite. **(B,C)** Modeled current densities at the end of conductive channels (fluid-filled fractures) at depths of 10 m **(B)** and 20 m **(C)**. Current density decays with depth away from the surface but increases again to fulgurite-forming magnitudes (> 10^5^ A/m^2^) in the simulated fluid-filled fractures.

A recent numerical model showed that lightning striking a planar half-space of rock can generate pressures in excess of 7 GPa and temperatures greater than 2000 K on the surface of the target rock^17^. To investigate whether or not sufficient electrical energy to form fulgurite can penetrate to a depth of several meters, we use the numerical modeling concept of ref. 12 and develop a simple model that; (1) uses the recently observed lightning currents from Kinmen Island (Fig. 1a) and, (2) contains low electrical resistivity pathways (i.e., simulated fluid-fractures) that are not connected to the surface (Fig. 8).

The results of the model show that the current density at the landing point on the surface is ~ 10^5.3^ A/m^2^, which then decays rapidly across the surface to reach non-fulgurite forming magnitudes (< 10^5.0^ A/m^2^) at c. 1 m distance from the landing point, consistent with the observed c. 1 m diameter of the fresh fulgurite observed on the outcrop surface (Fig. 8). The model also suggests that the current density decreases with depth, but less rapidly than it does along the surface. However, in the subsurface, the model indicates that the current density can increase to fulgurite-forming magnitudes (> 10^5.0^ A/m^2^) within the conductive layers. In the model setup used here, fulgurite-forming current densities are generated at c. 40 m from the impact point, even without connection of the high-conductivity channels to the surface (Fig. 8c).

## Discussion

Glass is commonly interpreted as a high-temperature product formed by melting and has previously been reported in rock fulgurites^8^, tectonic faults^18^, hypervelocity impacts^19^, and along the base of large landslides^20^. Our microstructural data documents the presence of glass in both surface and fracture-related fulgurite. While glass is a common component of rock fulgurite, our data also provide evidence for the presence of high-temperature and high-pressure features in both K-feldspar and plagioclase; namely plagioclase exsolution in K-feldspar, the change from a monoclinic to triclinic crystal structure in K-feldspar, a decrease in twin density in plagioclase as compared to the reference sample. The compositions and crystal structures of feldspars can vary continuously with changes in temperature and pressure. For example, monoclinic albite becomes triclinic at temperatures of ~ 1100 °C^21^, and monoclinic K-feldspar transforms to a triclinic phase at high pressures^22^. In addition, exsolution lamellae are produced when the host grains lose solubility during cooling to relatively low temperatures (> 1000 °C;^23,24^), or decompression to low pressures > 6 GPa^25^. Collectively, the observations of a phase transformation in K-feldspar, the widespread presence of exsolution lamellae in K-feldspar, and the reduced twin density in plagioclase from the Kinmen Island fulgurites can, therefore, be interpreted as resulting from high-temperature and high-pressure effects on K-feldspar during lightning strikes. This is consistent with the presence of glass, the high residual stresses preserved in K-feldspar grains (up to at least 1.57 GPa; Fig. 7). That there is a causal relation between these high-temperature and high-pressure features and lightening is supported by their absence in the reference granite sample collected from greater depths.

A numerical model showed that lightning striking a planar half-space composed of rock can generate local pressures of more than 7 GPa and local temperatures greater than 2000 K on the surface of the target material^17^, resulting in a form of shockwave. On this basis, we interpret the lightning-related high-temperature and high-pressure microstructures to be shock-like features, reminiscent of shock metamorphic textures produced during meteorite impacts^26,27^.

It is noted that despite the high temperatures required for melting, the quartz grains seem not appear to have undergone any type of recovery that would have subsequently destroyed any shock metamorphic textures (Supplementary Fig. S1). Theoretical studies suggest that the propagation of shock waves across heterogeneous interfaces may lead to longer duration of the same pressure and higher magnitude of temperature and promotes the kinetics of nucleation of the high-pressure polymorph^28,29^. As a result, the high pressure–temperature phase transitions in alkali feldspar can be sensitive to attribute to shock-induced phase transitions, which quartz grains are only brecciated, consistent with the observed low residual stresses (Supplementary Fig. S1). We therefore suggest that lightning strikes can give rise to a range of temperature- and pressure-dependent crystallographic reactions involving feldspars, although the specific nature of such reactions will require additional natural and experimental data to be collected and quantified.

The fracture-related fulgurite shows similar characteristics to the surface fulgurite: it contains glass (Fig. 5), K-feldspars that display a phase transformation, an abundance of exsolution lamellae in K-feldspar (Figs. 5 and 6), and a lower twin density in plagioclase compared to the reference sample. To our knowledge, this is the first time that such features have been described from the subsurface. The similarity in composition and textures between the two fulgurites suggests that the current density (and possibly the temperature and pressure conditions) in the fracture-related fulgurite may have been similar to that forming the surface fulgurite.

However, aspects of the organization and texture of the two fulgurites are different. In particular, the networks of altered glass in the fracture-related fulgurite are more chaotic and inject a highly fractured volume of quartz and feldspar grains at distances of up to several mm from the fracture surface (Fig. 5a–c). The melt that formed in the surface fulgurite, however, crystallized to form a thin glassy crust, and only locally is this glass injected along grain boundaries and cleavage planes in biotite (Fig. 3a,b). On the basis of our numerical modeling, we suggest that an increase in current density within fluid-filled fractures (i.e., an isochoric lightning channel; Fig. 8) could lead to a rapid increase in the temperature and volume of the fracture-filling fluid, resulting in simultaneous melting and brecciation of the host rocks. This process is broadly similar to the generation of thermal plasma and subsequent blasting and fragmentation produced in the laboratory^30–33^. Although the absolute pressure and temperature conditions under which the Kinmen Island fulgurites formed, the presence of glass and other microstructures in feldspar within the fracture-related example implies that lightning-induced thermal plasma is sufficient to melt granitic rocks at depth if the current density is able to travel along conductive fluid-filled fractures into the subsurface (c. 1000 ± 150 °C;^34^). This observation is supported by our numerical model.

Lightning can induce extreme pressure–temperature excursions, in some cases forming textures that resemble those produced by other extreme metamorphic events such as hypervelocity impacts. For example, a recent study of soil fulgurites showed that cubic ZrO_2_, which is formed under extreme pressure–temperature conditions and has been used as diagnostic criteria for meteorite impacts, can also be formed during lightning strikes^9^. The recognition of shock-induced minerals, textures, and glasses is commonly used to determine the presence of meteorite impacts (e.g.,^26,27^) and study the effects of fault-related friction melts (i.e., pseudotachylyte^35,36^). However, our work on fulgurites from Kinmen Island suggests that melts, high residual lattice stresses, and a range of high-temperature and high-pressure microstructures in feldspar can also form during lightning strikes on the surface and in fractures in the shallow subsurface. Thus, we recommend caution in the interpretation of low-level shock metamorphic features and suggest that, in certain circumstances, lightning-induced thermal plasma may be an alternative mechanism to generate such features, especially if the occurrences are spatially restricted and/or occur in narrow zones along fractures (i.e., meter-scale^37^).

Microstructural and mineralogical analysis of two rock fulgurites and a reference sample from Kinmen Island, Taiwan, indicates that a range of high-pressure and high-temperature features may form in fulgurites at the surface and along fractures in the shallow subsurface. Our observations show that both fulgurites contain: (i) a thin glassy layer (surface fulgurite) or a more complex network of glass injecting fractured host rock (fracture-related fulgurite), (ii) the presence of planar features in k-feldspar that are parallel to the (100) plane, (iii) a structural transformation in K-feldspar from monoclinic to triclinic, (iv) the presence of planar features in k-feldspar that are parallel to the (100) plane, (v) exsolution lamellae of plagioclase in K-feldspar, vi) a lower twin density in plagioclase compared to the reference sample, and (vii) heavily fractured quartz in the fracture-related fulgurite. Nevertheless, the absolute temperatures and pressures at which these features formed cannot be determined for the Kinmen Island fulgurites. Synchrotron Laue diffraction indicates that K-feldspar in the fulgurites records residual stresses of up to 1.57 GPa, well above the 0.38 GPa recorded in grains from the reference sample. Our work provides the first report of rock fulgurites developed along subsurface fractures and a description of the microstructures developed in this new type of occurrence. Our 1-D numerical modeling suggests that fracture-related fulgurite occurs because sufficiently high current densities can be carried by fluid-filled fractures to depths of at least several tens of meters. This broadens the range of near-surface environments in which extreme metamorphic processes can take place and also presents a detailed description of high-pressure and high-temperature microstructures that can be compared to those developed during other types of low-level shock metamorphism (e.g. meteorite impacts).

## Methods

### Fieldwork and lightning data

Fieldwork was performed on Kinmen Island on 11th June 2018. Two samples of rock fulgurite were collected from an outcrop near the summit of Mt. Taiwushan and are described in detail in “Discussion” below. As reference material, we obtained one sample of granitic gneiss from a borehole drilled through the Taiwushan Formation near the summit of Mt. Taiwushan. The reference sample was collected from a depth of 138 m. All three samples were cut into standard 30 µm-thick polished petrographic thin sections for examination using an optical microscope and scanning-electron microscope (“Results”). Additionally, the samples were studied using a focused ion beam transmission-electron microscope (“Discussion”) and synchrotron-based Laue Diffraction (“Methods”).

Lightning data were obtained from Weather Risk Explore Inc., which collects and processes data from the Taiwan Total Lightning Network (TTLN). The TTLN is part of the Earth Networks Total Lightning Network (ENTLN) that is continuously monitoring global lightning occurrence. There are more than 1,800 sensors covering over 100 countries around the world. Based on the ENTLN monitoring technology, a broad frequency ranging from 1 Hz to 12 MHz can be recorded. During lightning strikes, electromagnetic waves will radiate outward. By measuring the arrival time and amplitude of the electromagnetic waveform via the nearest sensors, the peak current of the return strike and the position of the strike can be obtained through time-of-arrival detection methods.

### Scanning electron microscope (SEM) and electron backscatter diffraction (EBSD) analysis

SEM imaging and EBSD analysis were carried out using a Zeiss Sigma VP Electron Microscope (FEG SEM) at the OMNI Centre at the University of Otago. Backscatter images were acquired on carbon-coated thin sections using an acceleration voltage of 15 kV and an aperture of 120 µm. Electron Dispersive Spectroscopy (EDS) analysis, used to determine the chemical composition of mineral phases, was performed with an HKL INCA Premium Synergy Integrated EDS/EBSD system (Oxford Instruments). For EDS analysis the following conditions were used: acceleration voltage of 15 kV, aperture of 120 µm and a working distance of 8.5 mm in high vacuum mode. EBSD diffraction patterns were collected with a Symmetry EBSD detector (Oxford instruments) on SYTON polished and carbon-coated thin sections using an accelerating voltage of 30 kV and an aperture of 300 µm resulting in a beam current of 100 nA. Crystallographic orientation maps, phase maps, and band contrast images were acquired using AZTEC software (Oxford Instruments). Post-acquisition data processing and analyses (e.g., crystallographic preferred orientation (CPO), misorientation profiles) were performed with Channel5 software.

### Focused ion beam-transmission electron microscopy (FIB-TEM)

We prepared the transmission electron microscopy (TEM) specimen by using a focused ion beam (FIB) system, FEI Helios 600i at National Taiwan University. A thin piece of the sample (10 μm by 4 μm) was cut by gallium ion beam in the FIB system and lifted off using an in-situ manipulator. The specimen was mounted on a Cu supporting washer, followed by further thinning of the specimen to < 100 nm. Bright-field TEM images were captured. Selected-area electron diffraction (SAED) patterns were collected from targeted regions of the fulgurites.

### In-situ synchrotron Laue diffraction analyses

Laue diffraction can reflect entire lattice planes from the crystal. The characteristics of the Laue pattern reveal the structure and orientation of the crystal, and the position displacement of diffraction spots between unstrained reference and experimental data can be used to calculate the deviatoric strain tensors (εij) in the sample. In this work, all the Laue diffraction patterns were automatically analyzed by using the software package XMAS^38^. A database of sanidine (AMCSD 0000312) and oligoclase (AMCSD 0010721) is used to calculate the strain tensor for fulgurites and the reference granite sample, respectively. After the index and calculation, six strain tensor components (ε11, ε22, ε33, τ12, τ13, τ23,) give the quantity and direction of principle and shear strain in the crystal. Negative values refer to compressive force, and positive values pertaining to tensile force. Anisotropic elastic stiffness constants (Cijkl) are applied into Hooke’s law σij = Cijkl εkl to estimate the deviatoric stress tensors (σij). An order-of-magnitude indicator of stress tensors was defined as von Mises equivalent stress^39^:

$${\upsigma }_{\mathrm{xy}}=\sqrt[2]{\frac{{{(\upsigma }_{11}-{\upsigma }_{22})}^{2}+{{(\upsigma }_{22}-{\upsigma }_{33})}^{2}+{{(\upsigma }_{33}-{\upsigma }_{11})}^{2}+6{({{\upsigma }_{12}}^{2}{{+\upsigma }_{13}}^{2}+{{{\upsigma }_{23}}^{2})}}}{2}}$$

The von Mises equivalent stress resolution depends on the shape of diffraction peaks and the deviation angle from index results. In this study, we have 0.2 GPa, 0.13 GPa, and 0.06 GPa errors for the surface fulgurite, fracture-related fulgurite, and reference granite sample, respectively.

Synchrotron experiments were carried out at the Taiwan Photon Source (TPS) Beamline 21A1 FORMOSA (FOcus x-Ray for MicrO-Structure Analysis) end-station at the National Synchrotron Radiation Research Center (NSRRC), Taiwan. FORMOSA is dedicated to white-/mono-beam Laue diffraction for structural analysis. For the above measurements, the spatial resolution can reach 80 × 80 nm. Therefore, the beamline utilizes a pre-shaped Kirkpatrick-Baez mirror pair to focus the polychromatic X-ray beam with energies ranging from 5,000 to 30,000 eV. The sample was navigated by an online real-time scanning electron microscopy (SEM) with a spatial resolution of 4 nm. All measurements were performed in vacuum. The specimen was mounted on a custom sample stage with a 45° pre-tilted angle relative to the incident X-ray beam. Diffraction patterns are collected using a high sensitivity hybrid pixel array detector (PAD, PILATUS3-X-6 M), located on the top of the focus point above the sample at 530 mm (this setup provides an angular resolution of < 0.018°). Diffraction patterns were analyzed using XMAS software^38^ to identify crystal phases and the related stress distributions.

### 1-D numerical modeling

We performed 1-D numerical modeling to investigate the depth to which the effects of lightning can potentially be recognized. Numerical modeling of electric current penetration was performed using COMSOL Multiphysics®. This software provides a numerical technique to simulate the electric field intensity and distribution using finite element methods. Calculations can be done with practical dimensions and material properties of various electric, half-space models for the ground. Using the AC/DC module of COMSOL Multiphysics®, we solved the following steady-state equations of electric field:

$$\begin{gathered} \nabla \cdot {\text{J}} = {\text{Q}}\_{\text{j}} \hfill \\ {\text{J}} = \sigma {\text{E}} + {\text{J}}\_{\text{e}} \hfill \\ {\text{E}} = - \nabla {\text{V}} \hfill \\ \end{gathered}$$

where J denotes the current density, Q_j the current source caused by lightning, σ the electrical conductivity of the half-space model for the ground, E the electric field, J_e the external current density and V the electric potential.

The model setup is that of a planar, homogenous rock half-space with a discretization of 1 cm. The half-space has an electrical resistivity of 1000 Ω-m and two 1-cm-thick conductive layers inside the rocks at depths of 10 and 20 m (Fig. 4a). The 1-cm-thick conductive layers have an electrical resistivity of 3 Ω-m (higher than seawater^40^) and are used to simulate fluid-saturated (composition of seawater) fractures that are not connected with each other. Because the dissipation of energy during a cloud-to-ground lightning pulse is uncertain (typically assumed to be about 1%^41^), this leads to an overestimation of the peak current density at the landing point. Increasing the width of the conductive layers results in lower current densities along them. This compensates for the higher current density that would occur along fractures if they were modeled with a width more appropriate for naturally occurring joints and fractures (c. 10 μm to 1 mm^42^). We assume that the injected lightning current intensity to the ground is 162 kA, which was observed in the 2018 cloud-to-ground lightning event on Kinmen Island.

## Supplementary Information

Supplementary Figure S1.

## Data Availability

All data are available in the main text or the supplementary materials.
