# Supplementary material for: Lightning-induced high temperature and pressure microstructures in surface and subsurface fulgurites
Source: Sci Rep. 2021 Nov 11;11:22031. doi: 10.1038/s41598-021-01559-x (PMC8586016; doi:10.1038/s41598-021-01559-x)
Supplement: Supplementary file 1 — Supplementary Figure S1. [file 41598_2021_1559_MOESM1_ESM.docx]

**Supplementary Materials**

**1. Synchrotron Laue diffraction on quartz adjacent to the analyzed K-feldspar**


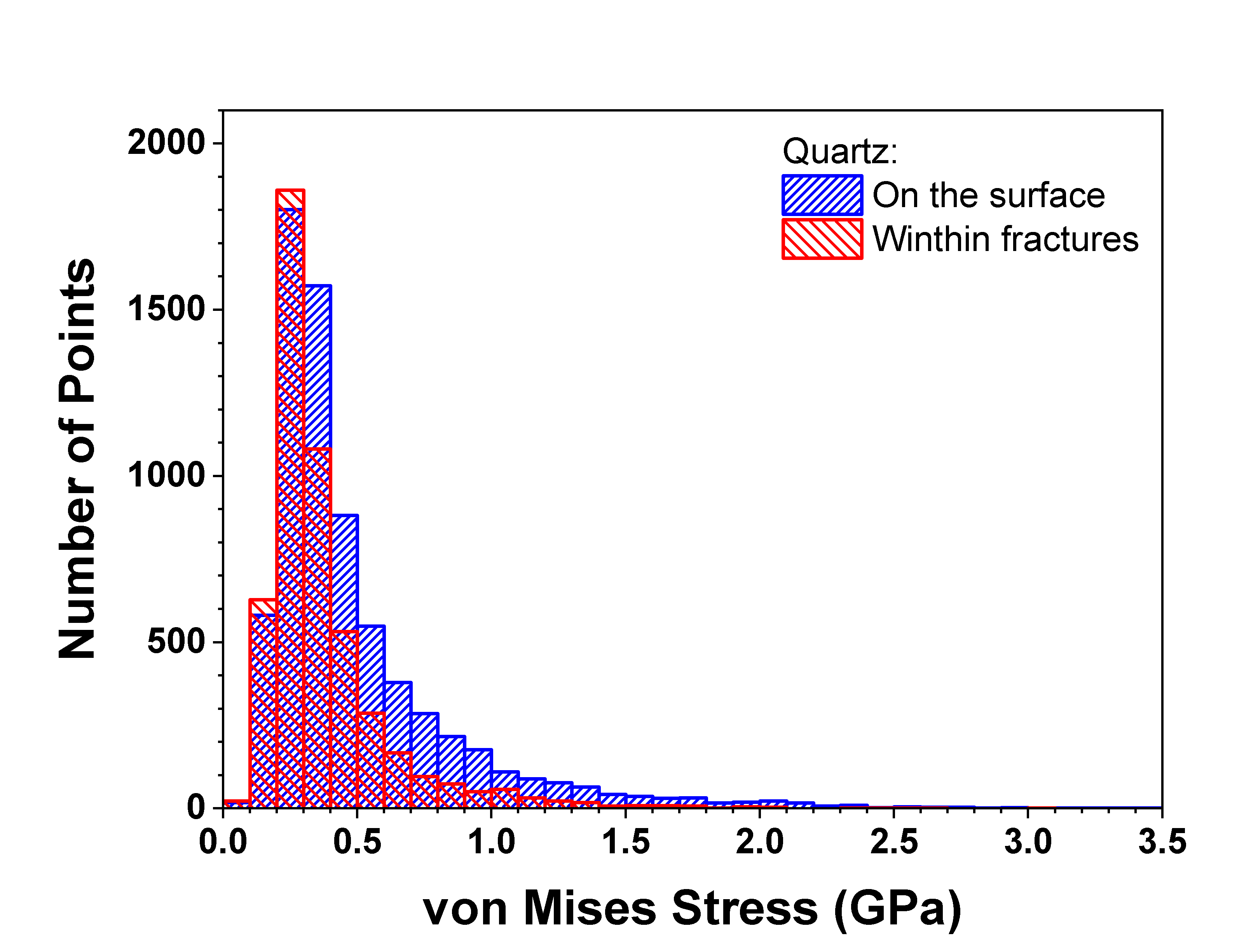


Figure S1. The statistics of residual stress from quartz grains adjacent to the analyzed K-feldspar from two fulgurites.
